# Editorial: Biotic and abiotic stresses on honeybee physiology and colony health

**DOI:** 10.3389/fphys.2023.1260547

**Published:** 2023-07-31

**Authors:** Zheguang Lin, Ming Zheng, Zhiguo Li, Ting Ji

**Affiliations:** ^1^ College of Animal Science and Technology, Yangzhou University, Yangzhou, China; ^2^ College of Animal Science (College of Bee Science), Fujian Agriculture and Forestry University, Fuzhou, China

**Keywords:** colony losses, pathogen and parasite, agrochemical, environmental change, nutritious diet

The honeybee, *Apis mellifera*, is a well-known economic eusocial insect and serves as the most critical pollinator that provides a key ecosystem service that underpins crop production and sustainable agriculture. Wild and managed honeybee colonies live in cave or non-cave nests and visit various flowering plants by foraging activities, during which they face a variety of biotic and abiotic threats considering the dense network of contacts among related nestmates and intercolonial individuals, chemical exposure, environmental pollutants, urbanization process, climate change, agricultural intensification, etc. A number of environmental and colonial stressors have considerable effects on honeybee individual health, colony development, and even colony survival. Consequently, colony losses have emerged as a crucial problem in global apiculture during the past few years.

The aim of this Research Topic “*Biotic and abiotic stresses on honeybee physiology and colony health*” in Frontiers in Physiology is to stimulate deep discussion and identify specific challenges regarding honeybee physiology when facing threats from inside and outside of the colony. This Research Topic has collected five scientific contributions from highly qualified research groups focusing on the potential impacts of various biotic and abiotic stressors on honeybee physiology and colony health.

The Earth’s environment is constantly changing, and species, including the honeybees, have to adapt to survive. Although insect individuals are poikilothermic, social insects can maintain the colony thermostasis as a superorganism. Shivering thermogenesis in honeybees has been shown to be dependent on octopamine signaling, but how the thoracic neuro-muscular octopaminergic system responds to cold stress is unclear. Kaya-Zeeb et al. found that the release of octopamine and the expression of octopamine receptors were maintained at a stable level in the organism. This mechanism facilitates honeybees to maintain endogenous thermogenesis and thus to respond rapidly to cold stress.

In addition to the effects of environmental changes, human activities, such as the application of agrochemicals, can also affect the honeybee health. Flumethrin is widely used to treat *A. mellifera* colonies against the ubiquitous ectoparasitic mites, and the negative effects of its residues on colony health has been recognized. Liu et al. investigated the potential toxicity of flumethrin on larvae and emerging adult bees during larval exposure by enzyme bioassays and transcriptome sequencing. As a result, a greater physiological harm was found to honeybee larvae and newly emerged adults when exposed to relatively higher concentration of this acaricide.

As a hot area of scientific research in recent years, non-coding RNAs (ncRNAs) have been proven to play important roles in biological processes. Abdelmawla et al. fed *Apis cerana* queens with *A. mellifera* royal jelly and found that nutritionally crossbred queens turned pale and yellow. Multiple differentially expressed ncRNAs were detected between nutritionally crossbred queens and *A. cerana* queens. Knockdown of key genes further confirmed that the body color of *A. cerana* could be changed by feeding *A. mellifera* diets, which might be the result of the expression alteration of non-coding RNAs and their related genes. Long non-coding RNAs (lncRNAs) are critical regulators across a broad range of biological functions in organisms. By infecting *A. mellifera* larvae at different ages with the fungus *Ascosphaera apis*, Ye et al. reported differential expression of hundreds of IncRNAs that potentially participated in the immune response of gut tissues by regulating the expression of neighboring genes or interacting with miRNAs.

Adequate nutrition is vital to the health of honeybees at all life stages, and pollen provides the major source of proteins and lipids. Corona et al. investigated the effects of pollen stress on honeybee immune response and the level of common bee viruses in the field. The expression of storage proteins was evidenced to be related to the division of labor, pollen restriction, and age, while genes involved in hormonal regulation presented higher expression levels in young foragers from colonies without pollen restriction. Intriguingly, pollen ingestion was positively correlated with higher titers of deformed wing virus but with lower titers of black queen cell virus. The authors concluded that behavior, followed by age and nutrition, greatly influenced gene expression and viral titers.

In the context of global insect and pollinator decline, honeybee health has long been a hot topic in apiology. These five impressive studies investigated the effects of biotic and abiotic stressors on honeybee physiology and colony health through different lenses including environmental temperature, agrochemicals, microorganisms, and nutrition. In the field, interactive and cumulative effects of multiple stressors generally occur and may plausibly be more detrimental than a single one, which merits further investigation and warrants colonial evaluation.

